# Gottfried Benn´s “brains” novella from 1916: implications for the philosophy of mind

**DOI:** 10.1186/s13010-025-00165-3

**Published:** 2025-02-19

**Authors:** Gunter Wolf

**Affiliations:** Jena University Hospital, Jena, Germany

**Keywords:** Humanities, Literature, Gottfried Benn, Philosophy of the mind, Physicalism in the body-mind discussion

## Abstract

Gottfried Benn (1886–1956) was a major 20th-century German physician-poet and essayist. He successfully completed his medical studies and worked across several hospitals and disciplines, including pathology, performing many autopsies. Later in life, Benn ran his own practice to treat skin and venereal diseases in Berlin for many years. Benn is not well-known in English-speaking countries and only a few of his poems have been translated into English so far. One possible reason for this may have been his initial enthusiasm for National Socialism (as documented in essays, see below), which led to a break with other authors of his generation who had to emigrate. His novella collection "Gehirne" (Brains), published in 1918, was a significant contribution to expressionist short prose. In this work, Dr. Rönne (Benn's alter ego) searches in vain for the soul and personality in the brain matter. This leads to increasing psychological instability and depression, and Rönne can no longer carry out his medical work. The purpose of this article is to familiarize the interested reader with Benn's biography and to analyze the novella in more detail, especially in terms of the concepts of the mind’s philosophy.

**Supplementary Information:**

The online version contains supplementary material available at 10.1186/s13010-025-00165-3.

## Introduction

Literature, as well as music and arts, have been used in the so-called "humanities" to teach medical students virtues such as integrity, compassion, empathy, care, and justice [1–4]. Often, but not always, doctors are presented as positive role models who exemplify such values [1]. However, I argued in 2006 that even negative role models, such as doctor Ferdinand Bardamu in Louis-Ferdinand Céline's novel "Journey to the End of Night", can teach important insights by examining the unprofessional, even criminal behavior of the protagonist [5].

There is a long tradition of physician-authors, especially in Germany, including Friedrich Schiller (1759–1805), Georg Büchner (1813–1837), Hans Carossa (1878–1956), and Alfred Döblin (1878–1957) [6]. In English-speaking countries, Arthur Conan Doyle (1859–1930), Silas Weir Mitchell (1829–1914), Anton Chekhov (1860–1904), William Carlos Williams(1883–1963), Samuel Shem (born 1944) and Neil Kurtzman (born 1936) are among many other well-appreciated writer-physicians [7, 8]. A not-so-well-known example of a physician-author outside of Germany is Gottfried Benn (1886–1956). He gained notorious fame with his expressionistic poems describing suffering and death in vivid, drastic, and disturbing, almost disgusting language [9–14]. The present contribution will briefly review Benn's unsteady life and focus on his 1916 novella "Gehirne" (Brains), in which the protagonist (likely Benn's alter ego) desperately tries to find mental processes, feelings, and the soul in the naked physical brain. This reductive material approach to the body-soul relationship has severe negative effects on the protagonist [15].

## Who was Gottfried Benn? A short biography

He was born on May 2, 1886 in Mansfeld, a small village in Brandenburg, Germany, approximately halfway between Hamburg and Berlin [9]. His father was the local Protestant pastor, a very devout and conservative person. Benn was born in the rectory, a building made of clay and beams from the seventeenth century, indistinguishable from a sheepfold. He grew up with the other village boys, ran barefoot until November, and herded cows. Benn had seven siblings: an elder sister, five younger brothers and a younger sister (born in 1901, who later also lived in Berlin like him). His father's regimen was strict, and Benn had to supervise his younger siblings at an early age. Benn was very close to his mother. He had a major dispute with his father in 1912 that caused irreversible damage to their relationship [10]. The reason was that his father had strictly forbidden Benn, who had just qualified as a doctor, from using morphine to alleviate the pain of his cancer-stricken mother. His father said her pain was God-given and not wanting to bear it was sinful. From 1896, the 10-year-old Benn attended the well-known Friedrich-Gymnasium in Frankfurt (Oder). He lived with other pupils in a boarding house. He learned ancient Greek and Latin and developed a close relationship with Greek antiquity and mythology. Benn was not an exemplary pupil, but the fact that he passed his school-leaving examination in 1903 at the age of 16 is not only an expression of his general intelligence but perhaps also of the efficiency of the school system. Benn actually wanted to study medicine, but his father forced him to pursue philology and theology in Marburg from September 1903. Benn hated his study subjects, despite the increasing popularity of Marburg philosophy, thanks to Neo-Kantians Hermann Cohen and Paul Natorp. Nevertheless, he began to write his first poems in Marburg. Eventually, realising that Marburg was too provincial, Benn moved to Berlin for the winter semester of 1904/5, where he continued to study philology and theology but soon switched to medicine. He studied at the academy for future military doctors as his father could not afford a civilian degree in medicine. Benn highly praised his medical training at the military academy; he interned in psychiatry at the Charité and passed the medical state examination in 1911, followed by his doctorate in 1912 [11].

He worked as a doctor in a pioneer battalion in Berlin, made the acquaintance of various writers and expressionist artists, and published his poems in a collection called Morgue. These poems, which thematize events in a morgue, provoked a scandal due to their drastic subject matter and casual language, instantly making the author famous as a representative of the newly emerging expressionist poetry. From 1913, Benn worked in various Berlin hospitals and carried out around 300 dissections. In 1914, he worked as a ship's doctor on a mail steamer and visited New York. After this, he was employed in a lung sanatorium in the Fichtelgebirge. In the same year, he married his first wife, Edith. He was immediately drafted at the start of the war in 1914. From 1915 to 1917, Benn was a senior physician in the military governorate in Brussels (Belgium) in a hospital for prostitutes. In September 1915, his daughter Nele (his only child) was born.

The prose "Gehirne" (Brains) with the protagonist Dr. Rönne (see below) was published in the brochure series "Der jüngste Tag" by publisher Kurt Wolff in Leipzig in 1916. In November 1917, Benn set up his practice as a specialist for skin and venereal diseases in Berlin and ran it until 1935. Although Benn was married, he constantly had intimate relationships with other women, some of whom were much younger. His motto was: "Good direction is better than loyalty." Edith died at the age of 44 following a gall bladder operation in 1922, and Nele was adopted by a Danish couple without children [12].

For around a year and a half from Adolf Hitler's seizure of power on January 30, 1933, until the Röhm Putsch on June 30, 1934, Benn was an enthusiastic supporter of the National Socialists and also campaigned for them in publications, radio speeches, and his work at the Academy of Arts. Here, he signed an unconditional declaration of loyalty to the Nazi regime. As a result Thomas Mann, Heinrich Mann, Alfred Döblin, Bertolt Brecht and some others resigned from the academy and many went into exile. During this time, Benn also published essays in which he strongly supported the biologistic racial ideology of the Nazis (e.g., "Züchtung" Breeding in 1933). Moreover, Benn supported in an essay directly the Nazi regimen:“Führer that ist he principle of creativity. He embodies not only responsibility. Danger and decisiveness, but also the irrational core of the will of history, which has only now become manifest through him “ ([9], p. 257]) However, Benn was soon forced out of the academy by party members, and his expressionist literature was massively criticized in public. After the Röhm Putsch in 1934, Benn himself wanted nothing more to do with the Nazis. He was disillusioned and probably also offended that his pro-Nazi commitment had not been recognized. In retrospect, Benn's brief enthusiasm for the Nazis has rightly been heavily criticized,. But he was probably more a supporter of a conservative revolution coupled with a fundamental criticism of civilization than a truly avowed National Socialist. On April 1, 1935, Benn gave up his practice in Berlin and returned to the army as a senior medical officer (Army Medical Inspection, Hannover).

On his 60th birthday in 1936, "Ausgewählte Gedichte" (Selected Poems) was published, and Benn was massively defamed by the SS. In 1937, Benn was transferred to Berlin and became responsible for medical reports for military service. In 1938, he married Herta von Wedemeyer, 21 years his junior (he later said that he needed someone to run the household, indicating it was not a love match), and in the same year, he was expelled from the Reichsschrifttumskammer [13].

After the start of the war in 1939, Benn was promoted to senior field doctor. He worked temporarily at the Wehrmacht High Command in Berlin. In 1943, Benn was transferred to Landsberg an der Warthe (now Zielona Góra, Poland). He fled to Berlin in January 1945, while his second wife committed suicide. Benn reopened his practice and married his third wife, Ilse, in 1946. In 1949, various works written during the war were published, and Benn's fame grew. In 1951, Benn was awarded the prestigious Georg Büchner Prize for Literature in Darmstadt indicating that his work was appreciated by the literature mainstream after the war. In 1953, he gave up his art practice and was awarded the Federal Cross of Merit, first class. He died of cancer of unknown primary in 1956, in the 70th year of his life ([For more details see [14, 15]).

## Benn's "Gehirne" (Brains) with Dr. Rönne as the protagonist from 1916

### In what literature category can Benn be classified?

As a practicing dermatologist and venereologist, Benn's medical background deeply influenced his literary work. His clinical perspective on the human body and its ailments shaped his poetry's imagery and themes to a large extent. This can be seen in his unflinching portrayal of physical decay and his scientific, almost detached approach to human suffering. Benn's early poetry, including his influential collection "Morgue" (1912), is a prime example of expressionism, that is characterized by a focus on the inner experiences and emotions of individuals, often conveyed through intense, dramatic, and sometimes chaotic imagery and language. An example called „A lovely childhood “ ([9], S,43]) is:

The mouth of a girl who had lain long amongst water reeds.

Looked quite gnawed away.

When her chest was openend up, the gulletr was found tob e foll of holes.

And then, in the cavity below the diaphragm,

a nest of young rats was discovered.

One little sister lay dead,

the others were nourishing themselves on the girl´s liver and kidneys,

drinking her cold blood, and ha enjoyed here a lovely childhood.

But sweet and swift came their end too:

The whole lot were thrown into the water.

Oh, how their little snouts quicked.

His poems from this period are noted for their graphic, often macabre, depictions of the human body and illness, reflecting his background as a physician. Moreover, Benn's work often grapples with themes of nihilism and existentialism. His poetry and prose frequently explore the futility of existence, the decay of the human body, and the absence of any higher meaning or purpose in life. This philosophical outlook can be seen in his later works, where he delves into the isolation and alienation of the modern man.

## Dr. Rönne (Benn's alter ego) in Brains

**Fig. 1:**
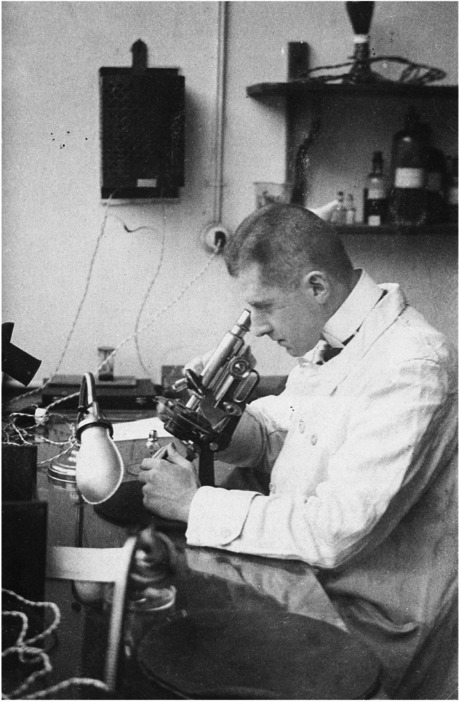
Gottfried Benn in 1916 when *Gehirne* was first published

Although some poems, essays, and select prose have been translated into English [16], the majority of Benn's work is only accessible in German. To the best of my knowledge, "Gehirne" has not been translated into English. A few single paraphrases are cited in the excellent and only English biography of Benn by Martin Travers [15]. Gehirne" from 1916 comprises six chapters: Gehirne (Brains), Die Eroberung (The Conquest), Die Reise (The Journey), Die Insel (The Island), and Der Geburtstag (The Birthday), all describing life episodes and experiences of the young physician Dr. Rönne. For space constraints, I will focus only on the first chapter, Gehirne (Brains), which gave the novel its name. The original German version of Gehirne can be found on the internet [17]. I translated the first part of Gehirne (see Supplement 1): Benn as a doctor in Brussels serves as a role model for Doctor Werff Rönne, the protagonist of the novella. Benn, as well as his alter ego Rönne, are approximately 30 years old at the time of the events. Figure 1 shows Benn sitting near a microscope in a laboratory in 1916, the year of the novella. The opening passage of the novella with the train journey may be based on Benn's memories of his return journey from Bischofsgrün in the Fichtelgebirge, where he had replaced the head physician of a pulmonary clinic in the summer of 1913. Gehirne begins with Rönne sitting in the train and traveling north to take over as head physician of a hospital for a few weeks. He was previously employed at a pathology institute and had dissected many corpses. Rönne thought he had spent this time doing nothing. As he’s en route to his new place of work, the landscape appears to him like an impressionist painting, as the movement of the train prevents him from precisely grasping the objects; the contours dissolve and the sensory perception is literally blurred. He sees vines, poppies, and roses, all intoxicating plants that impair the function of the cerebrum and make it difficult to act rationally. Rönne's later psychological problems are already apparent here. He decides to record his experiences in writing and describes how he autopsied over 2000 corpses, which exhausted him greatly. Rönne turns his scientific-analytical gaze on himself and experiences an increasing ego disintegration with the dissolution of his personality and a progressive loss of reality [18–20]. Rönne is characterized by complete passivity and can no longer perceive his environment rationally or treat patients. He falls into a state of complete apathy and despondency, while his psyche is rather hyperactive. Rönne repeatedly looks at his hands and asks himself how the brain functions in detail and whether there is a connection to the wearer's identity. However, he does not find one, because he remains attached to his purely scientific view. Rönne ultimately sees his patients as an accumulation of various symptoms and regards himself as a disintegrating symptom. Rönne muses that he cannot find a point of support for existence in the brain; he is faced with nothing and becomes a nihilist. The mental decay continues, and the original chief physician has to be recalled, as Rönne can no longer perform his clinical tasks. The central role of the brain is also reflected in the section of the novella titled Die Insel (The Island). Here, Benn himself completely negates the soul, with which Descartes still knew what to do, and writes: "While Cartesius had still assumed the pineal gland to be the seat of the soul, since its exterior might resemble the finger of God— yellowish, elongated, mild and yet threatening — brain physiologists had determined when sugar appeared in the urine when indigo appeared, even when saliva flowed correlatively. The accompanying character of the feelings to the sensations was recognized by psychology, the general value of the defense against the harmful to which they were entitled was determined in precise curves, and the readability of the individual differences was complete. With the renewal of Berkeley's ideas, epistemology concluded to help a panpsychism achieve a breakthrough, which assigned the real rank of condensed concepts in the meaning of a sexually emphasized environment for the purpose of convenient species preservation." ([17] translation by GW). The brain, hence, becomes the absolute superorgan.

The novella thematizes the thin line between sanity and madness. Rönne's obsession with the human brain and his clinical perspective on life lead him into a state of mental instability. The brain symbolizes rationality and scientific thinking. At the same time, however, Rönne's experiences show that excessive rationality can turn into madness. Through Rönne, Benn shows the existential emptiness and search for meaning in a dehumanized world. Rönne's experiences and inner monologue illustrate the existential crisis and the question of the purpose of life. Benn describes in "Gehirne" the brain in mechanical and clinical terms, criticizing the dehumanization and objectification of human beings in modern medicine and society. It is assumed that Rönne's stories reflect Benn's own experiences with drugs such as cocaine. Benn´s consume of drugs has been documented [9, 12, 13, 19].

The great significance of the Rönne novellas within Benn’s oeuvre as a whole is not the least due to the fact that he already uses central motifs there that he would later take up again and again or that run through his entire oeuvre, such as the brain theme in the field of tension between the critique of reason and, on the other hand, the provocative loss of rationality and identity up to and including madness. The consequences are loss of identity, depression, and socio-coldness [15, 18].

### What is the philosophy of the mind?

The philosophy of the mind explores the nature, origin, and functioning of the mind [21–23]. It addresses fundamental questions about mental phenomena and their relationship to the physical world, particularly the brain. Central to this field is the relationship between brain function and consciousness, as well as their interconnection. It also examines whether there are subjective and qualitative aspects of conscious experience (so-called qualia) and how mental states, including beliefs, desires, and sensations may relate to physical states, such as brain processes and neural activities. A comprehensive review of the philosophy of the mind is beyond the scope of this article. Interested readers are referred to excellent further references [21–23]. Broadly, there are two main concepts: dualism and monism. Dualism suggests that the mind and body are distinct and separable. René Descartes (1596–1650) is one of the most famous proponents of dualism, positing that the mind is a non-physical substance. He strictly distinguished between the mind and body, which he saw as two different kinds of substances. The mind is a non-extended, thinking substance (res cogitans), while the body is an extended, non-thinking substance (res extensa). According to his theory, the two components interact in the pineal gland. In contrast, monism posits that there is only one fundamental kind of substance or principle that constitutes reality, either physical or mental. The assumption that only mental or spiritual events are real is called idealism, and was proposed by George Berkeley (1685–1753). However, this view is almost no longer represented today. Although there are other concepts, such as neutral monism, physicalism (materialism) plays a major role in contemporary philosophy of the mind. This view holds that only physical substances exist, and mental states are physical states. This perspective often aligns with contemporary neuroscience and the belief that all mental processes can be reduced to brain (neuronal) processes. An absolute reduction of mental properties to brain processes can also be found in the work of the late neuroscientist Gerhard Roth (1942–2022), who wrote a book titled "How the Brain Makes the Soul". It may be just a coincidence that this book was published by the same publisher (Klett-Cotta) responsible for the first complete edition of Benn's works in 1956. Roth explains: "There can be no reasonable doubt that the brain produces the soul, and that it does so at very different levels of neuronal activity, from the processes at the synapses to the interactions of the whole brain with the body and the environment." ([24] translation by GW).

In his detailed analysis, Thomas Gann points out that Benn had a keen interest in psychiatry and even wrote a small contribution to the history of psychiatry in 1910 [25, 26]. Based on the work of Wilhelm Griesinger, it was believed at the time that psychiatric illnesses could be anatomically assigned to specific regions of the brain [26]. Benn adopted this concept and had Dr. Rönne search in vain for consciousness in the anatomical structures of the brain [17].

Dr. Rönne, who had carried out many dissections and held several thousand brains in his hands, searched in vain for the soul and the origin of personality in these brains. Rönne failed to solve this reductive scientific physical approach to the body-soul problem and increasingly took refuge in a fantasizing world, which he attributed to external sensory impressions. However, this led to a progressive disintegration of the ego, resulting in apathy, dejection, and depression. His turn to physicalism ultimately destroyed his soul [27].

## Conclusion

Thomas Fuchs (*1958) is a prominent German psychiatrist and philosopher, recognized for his work in phenomenology, psychopathology, and philosophy of the mind. He holds the position of Karl Jaspers, professor of philosophy and psychiatry at the University of Heidelberg. Fuchs wrote in the prologue of his book "The Brain: A Relational Organ" regarding the Brains novella: "The crisis of the young doctor Rönne results from an existential paradox: He himself, the observer, researcher, and thinker, seems to be nothing more than an object of his studies, namely a lump of gray matter that follows its own laws and has nothing to do with the human world. And yet Rönne's psychological crisis is ultimately only based on a mystification to which he is just as subject as many neuroscientists today: for it is not the brain that thinks. What Rönne holds in his hands, or what the brain researcher sees on his tomograms today, is not the seat of the soul, not the person itself, and not even its only carrier organ." ([28] translation by GW).

In the corpus callosum alone, there is an information traffic of 200 to 6000 billion events per second. It is obvious that researchers will not be able to provide an exact description of certain brain functions in the foreseeable future. For the neurosciences and for our understanding of behavior and brain functions, it follows that even if the brain functions deterministically, in its complexity it can never be completely understood. The paradox is that for physical reasons, a physical description of the mental will not succeed [29].

In contrast, Fuchs and others have developed a concept in which the brain is an organ of the living being, a non-reductive philosophy between the mind and brain involving an ecological, embodied, as well as enactive perspective. Fuchs sees the body and mind as corresponding fields. By this, he understands a holistic, non-reductionist concept of the whole organism, which sees itself as a self-regulating whole from the individual brain cell to the complex body. Fuchs explains this tiered structure of the organism from basic biochemical/physical processes at the cellular level via organs to the integrative body in detail using the example of the brain at a micro, meso, and macro level with circular top-down and bottom-up relationships [28, 30]. His approach may be described as holistic phenomenological anthropology. I am certain that Dr. Rönne would have prevented his own madness if he had known this philosophy.

## Supplementary Information

Supplementary Material 1. Supplement 1: The first part of *Gehirne* translated into English by the author of this contribution.

## Data Availability

No datasets were generated or analysed during the current study.
