# Supplementary material for: Gottfried Benn´s “brains” novella from 1916: implications for the philosophy of mind
Source: Philos Ethics Humanit Med. 2025 Feb 19;20:3. doi: 10.1186/s13010-025-00165-3 (PMC11837678; doi:10.1186/s13010-025-00165-3)
Supplement: Supplementary file 1 — Supplementary Material 1. Supplement 1: The first part of Gehirne translated into English by the author of this contribution. [file 13010_2025_165_MOESM1_ESM.docx]

BRAINS

Rönne, a young doctor who had previously done a lot of dissections, was driving through southern Germany towards the north. He had spent the last few months doing nothing; he had been employed for two years at a pathological institute, which meant that about two thousand corpses had passed through his hands without reflection, and that had exhausted him in a strange and unexplained way.

Now he sat on a corner seat and looked at the road ahead: it was through wine country, he thought to himself, fairly flat, past fields of scarlet, smoking with poppies. It's not too hot; a blue floods through the sky, damp and blown up by shores; every house is leaning against roses, and some are completely sunken. I want to buy myself a book and a pen; I want to write down as much as I can now so that it doesn't all flow down like this. I've lived for so many years and everything has sunk. When I started, did it stay with me? I no longer know.

Then, in many tunnels, the eyes were on the verge of catching the light again; men working in the hay, bridges made of wood, bridges made of stone; a town and a wagon over mountains in front of a house.

Verandas, halls and sheds, on the height of a mountain, built into a forest - this is where Rönne wanted to stand in for the chief physician for a few weeks. Life is so almighty, he thought, this hand will not be able to undermine it, and looked at his right hand.

There was no one in the grounds except staff and patients; the hospital was high up; Rönne felt solemn; surrounded by his solitude, he discussed official matters with the nurses in a distant and cool manner.

He left everything to them to do: turning the levers, fixing the lamps, driving the motors, illuminating this and that with a mirror - it did him good to see science broken down into a series of operations, the coarser ones worthy of a blacksmith, the finer ones of a watchmaker. Then he himself took his hands, passed them over the X-ray tube, moved the mercury of the quartz lamp, widened or narrowed a slit through which light fell on a back, pushed a funnel into an ear, took absorbent cotton and left it in the auditory canal, and immersed himself in the consequences of this operation on the owner of the ear: how ideas of helper, healing, good doctor, general confidence and joy of the world were formed, and how the removal of fluids became interwoven with the soul. Then came an accident and he took a small wooden board padded with absorbent cotton, slipped it under the injured finger, wrapped a starch bandage around it and thought about how this finger had been injured by jumping over a ditch or an overlooked root, by wantonness or carelessness, in short, how deeply connected with the course and destiny of this life it seemed to be broken, while he now had to care for it like a distant and runaway one, and he listened into the depths to see how a distant voice could be heard at the moment when the pain set in.

One morning he sat shaken in front of his breakfast table; he felt so deeply: the chief physician would be away, a deputy would come, get out of this bed at this hour and take the bread roll: you think you are eating and breakfast is working on you. Nevertheless, he continued to ask questions and give orders; he tapped one finger of his right hand on one of his left, then a lung stood underneath it; he stepped up to the beds: good morning, what is your body doing? But it could happen now and then that he would walk through the halls without properly questioning each individual, be it about the number of his coughs or the warmth of his bowels. When I walk through the halls - this preoccupied him too deeply - I fall into two eyes each, am noticed and considered. I am connected with friendly and serious objects, perhaps a house takes me in that they long for, perhaps a piece of tanning wood that they once tasted. And I also once had two eyes that ran backwards with their gazes; yes, I was there: unquestioning and collected. Where did I come from? Where am I? A small flutter, a drift.

He wondered when it had begun, but he no longer knew: I walk through a street and see a house and remember a castle that was similar in Florence, but they only touch each other with a glow and are extinguished.

Something weakens me from above. I no longer have any support behind my eyes. The room undulates so endlessly; once it flowed in one place. The bark that carried me has crumbled.

Often, when he returned to his room from such walks, he would turn his hands back and forth and look at them. And once a sister observed how he touched them, or rather how he walked over them as if he were testing their air, and how he then placed the slightly bent palms, open at the top, together on the little fingers, and then moved them towards and away from each other as if he were breaking open a large, soft fruit or prying something apart. She told the other sisters, but no one knew what it meant. Until it happened that a large animal was being slaughtered in the asylum. Rönne came over, apparently by chance, when the head was opened, took the contents in her hands and bent the two halves apart. Then it flashed through the sister's mind that this was the movement she had observed in the corridor. But she couldn't make the connection and soon forgot about it.

Rönne, however, walked through the gardens. It was summer; otter tongues swayed the blue sky, the roses bloomed, sweetly beheaded. He felt the urge of the earth: up to his soles, and the swelling of the forces: no longer through his blood. But he walked mainly on paths that lay in the shade and those with many benches; often he had to rest from the unrestrained light, and he felt exposed to a breathless sky.

Gradually he began to perform his duties only irregularly; but especially when he was supposed to speak to the steward or the matron about any subject, when he felt that it was now time for him to make a statement on the subject in question, he literally collapsed. What should one say about an event? If it didn't happen that way, it would happen a little differently. The place would not remain empty. But he just wanted to look quietly in front of him and rest in his room.

But when he lay, he did not lie like one who had come only a few weeks ago, from a lake and over the mountains; but as if he had grown up with the place on which his body now lay, and had been weakened by long years; and something stiff and waxy was long about him, as if taken from the bodies that had been his companions.

In the days that followed, too, he occupied himself much with his hands. The nurse who waited on him loved him very much; he always spoke to her so imploringly, although she did not quite know what it was all about. He often began a little scornfully: he knew these strange objects, his hands had held them. But he immediately fell back: they lived in laws that were not ours and their fate was as foreign to us as that of a river on which we sail. And then his gaze was completely extinguished in one night: they were twelve chemical units that had not come together at his command and would separate without asking him. Where should one go then? It would only blow over them.

He no longer faced anything; he no longer had any power over the room, he once said; he lay almost continuously and hardly moved.

He locked his room behind him so that no one could rush in on him; he wanted to open it and face it calmly.

He ordered hospital trolleys to drive back and forth on the country road; he had observed that it made him feel good to hear trolleys rolling: it was so far away, it was like before, it was going to a foreign city.

He always lay in one position: stiffly on his back. He lay on his back in a long chair, the chair was in a straight room, the room was in the house and the house was on a hill. Apart from a few birds, he was the highest animal. So the earth carried him quietly through the ether and past all the stars without shaking.

One evening, he went down to the lying-in halls; he looked along the deckchairs, how they were all quietly awaiting recovery under their blankets; he looked at them as they lay there: all from home, from sleep full of dreams, from evening homecomings, from songs from father to son, between happiness and death - he looked along the hall and went back.

The head doctor was called back; he was a friendly man, he said that one of his daughters was ill. But Rönne said: "You see, in these hands of mine I held them, a hundred or a thousand of them; some were soft, some were hard, all very melting; men, women, crumbly and full of blood. Now I always hold my own in my hands and must always search for what is possible with me. If the forceps had pressed a little deeper into the temple here . . .? If I had always been struck over a certain part of the head . . .? What is it with brains? I always wanted to fly up like a bird from the gorge; now I live outside in the crystal. But now please clear the way for me, I'm swinging again - I was so tired - on wings this walk goes - with my blue anemone sword - in midday fall of light - in debris of the south - in disintegrating clouds - atomizations of the forehead - digressions of the temple.

Translation by G:W.
